# Comparison of Ceftolozane-Tazobactam and Ceftazidime-Avibactam in the Treatment of Multidrug-Resistant/Difficult-to-Treat Resistant *Pseudomonas aeruginosa* Infections: A Systematic Review and Meta-analysis

**DOI:** 10.1093/cid/ciag194

**Published:** 2026-03-18

**Authors:** Milo Gatti, Riccardo De Paola, Beatrice Giorgi, Federico Pea

**Affiliations:** Department of Medical and Surgical Sciences, Alma Mater Studiorum, University of Bologna, Bologna, Italy; Clinical Pharmacology Unit, IRCCS Azienda Ospedaliero-Universitaria di Bologna, Bologna, Italy; Clinical Pharmacology Unit, IRCCS Azienda Ospedaliero-Universitaria di Bologna, Bologna, Italy; Specialisation School of Clinical Pharmacology and Toxicology, Alma Mater Studiorum, University of Bologna, Bologna, Italy; Clinical Pharmacology Unit, IRCCS Azienda Ospedaliero-Universitaria di Bologna, Bologna, Italy; Department of Medical and Surgical Sciences, Alma Mater Studiorum, University of Bologna, Bologna, Italy; Clinical Pharmacology Unit, IRCCS Azienda Ospedaliero-Universitaria di Bologna, Bologna, Italy

**Keywords:** ceftolozane-tazobactam, ceftazidime-avibactam, MDR/DTR *Pseudomonas aeruginosa*, clinical cure, resistance development

## Abstract

**Background:**

Ceftolozane-tazobactam and ceftazidime-avibactam are considered first-line treatment for multidrug-resistant (MDR) and/or difficult-to-treat resistant (DTR) *Pseudomonas aeruginosa* infections. Conflicting results in terms of clinical outcome and resistance development emerged from real-world comparative studies. We performed a systematic review with meta-analysis to compare ceftolozane-tazobactam and ceftazidime-avibactam in the treatment of MDR/DTR *P. aeruginosa* infections.

**Methods:**

Two authors independently searched PubMed-MEDLINE and Scopus databases from inception to 18 November 2025 to retrieve studies comparing ceftolozane-tazobactam versus ceftazidime-avibactam in the management of MDR/DTR *P. aeruginosa* infections. Clinical cure was set as the primary outcome, whereas 90-day resistance development, microbiological failure, and the 30-day mortality rate were set as the secondary outcomes. Meta-analysis was performed by pooling the odds ratios (ORs) retrieved from studies providing adjustment for confounders by means of a random-effects model with the inverse variance method. The risk of bias in the included studies was independently assessed using the RoB 2.0 and the ROBINS-I tools.

**Results:**

Among a total of 1193 articles screened, 6 observational studies were retrieved and included (n = 1161; 644 receiving ceftolozane-tazobactam vs 517 receiving ceftazidime-avibactam). Ceftolozane-tazobactam showed a higher clinical cure rate than ceftazidime-avibactam (n = 4; OR, 1.82 [95% confidence interval, 1.10–2.99]; *I*^2^ = 25.3%). No difference emerged in terms of microbiological failure (n = 2; OR, 0.66 [95% confidence interval, .36–1.19]; *I*^2^ = 21.9%), 90-day resistance development (n = 3; OR, 1.11 [.51–2.42]; *I*^2^ = 50.0%), or 30-day mortality rate (n = 4; OR, 1.00 [.89–1.12]; *I*^2^ = 0.0%).

**Conclusions:**

Our meta-analysis showed that, after appropriate adjustment for confounders, ceftolozane-tazobactam could grant a higher clinical cure rate than ceftazidime-avibactam in the treatment of MDR/DTR *P. aeruginosa* infections.

## Teaser

Real-world comparative studies comparing ceftolozane-tazobactam and ceftazidime-avibactam in multidrug-resistant/difficult-to-treat resistant *Pseudomonas aeruginosa* infections had conflicting results in terms of clinical outcome. Our meta-analysis showed that, after appropriate adjustment for confounders, ceftolozane-tazobactam enabled a higher clinical cure rate.

*Pseudomonas aeruginosa* is among the pathogens most frequently causing healthcare-associated infections in immunocompromised or critically ill patients and may account for a remarkable increase in morbidity and mortality rates [1, 2]. The most common types of infection caused by *P. aeruginosa* in hospitalized patients are hospital-acquired pneumonia, ventilator-associated pneumonia, bloodstream infections (BSIs), urinary tract infections, and surgical site infections [2]. Unfortunately, *P. aeruginosa* shows an intrinsic ability to rapidly develop resistance to first-line antibacterial agents by means of several underlying mechanisms. This may make the choice of an appropriate treatment for *P. aeruginosa* infections extremely challenging [2, 3]. In addition, the adoption of several different definitions of the phenotypic resistance of *P. aeruginosa* (ie, multidrug-resistant [MDR] or difficult-to-treat resistant [DTR]) [4, 5] may be confusing, making it even more complicated to select the most appropriate agent [6].

Ceftolozane-tazobactam and ceftazidime-avibactam are 2 β-lactam–β-lactamase inhibitor combinations showing excellent activity against MDR and/or DTR *P. aeruginosa* infections [7]. In vitro testing of different large collections of *P. aeruginosa* isolates showed that >90% of the strains were fully susceptible to both combinations, irrespective of being resistant to piperacillin-tazobactam, antipseudomonal cephalosporins, and carbapenems [8, 9]. Currently, both of these agents are recommended by the Infectious Diseases Society of America guidance as first-line treatment for severe MDR or DTR *P. aeruginosa–*related infections [10]. Indeed, from a pharmacological standpoint it should not be overlooked that the 2 agents may exhibit some differences, possibly affecting clinical outcomes.

The intrinsic activity against *P. aeruginosa* may differ according to the microbiological spectrum [1, 3, 11, 12]. Ceftolozane showed valuable activity against those strains overexpressing AmpC or defecting OprD porin [3, 13, 14]. In addition, in terms of pharmacokinetic/pharmacodynamic (PK/PD) targets, ceftolozane does not need tazobactam to restore activity against DTR *P. aeruginosa*, irrespective of several resistance mechanisms. Conversely, ceftazidime-avibactam needs appropriate targeting of avibactam to restore ceftazidime activity against DTR *P. aeruginosa*. Avibactam is essential for preventing ceftazidime from degradation by both wild-type *Pseudomonas*-derived cephalosporinase and other types of β-lactamases [1–3, 11, 12]. Unfortunately, in patients without renal dysfunction the right proportion between ceftazidime and avibactam may be affected by faster renal elimination of avibactam than of ceftazidime, leading to a disproportion between the 2 compounds [15]. Consequently, the proper joint PK/PD target of ceftazidime-avibactam may be more difficult to attain in patients with normal renal function or augmented renal clearance [15]. Overall, these pharmacological differences between ceftolozane-tazobactam and ceftazidime-avibactam could theoretically lead to differences in the treatment of MDR/DTR *P. aeruginosa* infections.

Several studies have compared ceftolozane-tazobactam with ceftazidime-avibactam in the treatment of MDR and/or DTR *P. aeruginosa* infections. Unfortunately, the emerged findings were conflicting in terms of both clinical efficacy and resistance development, raising some key questions for today's clinicians [3]. Based on these assumptions, a systematic review with meta-analysis was performed to compare the clinical efficacy of ceftolozane-tazobactam versus ceftazidime-avibactam in the management of documented MDR and/or DTR *P. aeruginosa* infections.

## METHODS

The systematic review comparing clinical efficacy of ceftolozane-tazobactam versus ceftazidime-avibactam in the management of documented MDR and/or DTR *P. aeruginosa* infections was registered in the PROSPERO database (CRD420251236181) and conducted according to the Preferred Reporting Items for Systematic Review and Meta-Analyses (PRISMA) guideline [16].

### PICO Framework

Defined according to the PICO framework (ie, Population, Intervention, Comparator, and Outcome), the study population included patients with documented infections caused by MDR or DTR *P. aeruginosa*; the intervention, targeted therapy with ceftolozane-tazobactam; the comparator, targeted therapy with ceftazidime-avibactam; and the outcome, clinical cure.

### Search Strategy

The PubMed-MEDLINE and Scopus databases were searched independently by 2 authors (M. G. and R. D. P.) from inception to 18 November 2025. The specific search string was (“Pseudomonas” OR “Pseudomonas aeruginosa” OR “multidrug resistant Pseudomonas aeruginosa” OR “MDR Pseudomonas” OR “DTR Pseudomonas aeruginosa” OR “difficult-to-treat resistant Pseudomonas aeruginosa”) AND (“ceftolozane-tazobactam” OR “ceftolozane”) AND (“ceftazidime-avibactam” OR “avibactam”). No language restriction was set. The reference lists of the retrieved studies were screened independently by 2 authors (M. G. and R. D. P.) to identify articles potentially fulfilling the inclusion criteria, paying attention to duplicate removal.

### Study Selection

Only randomized controlled trials (RCTs) and/or observational studies comparing the clinical efficacy of ceftolozane-tazobactam versus ceftazidime-avibactam in the treatment of adult patients with documented MDR/DTR *P. aeruginosa* infections were included. The MDR/DTR status of *P. aeruginosa* was defined based on the original definition set in each included study. Exclusion criteria were the absence of quantitative data concerning the selected outcomes, of a comparator group, or of any analysis providing adjustment for confounders.

Clinical cure was set as the primary outcome, and it was defined based on the original definition set in each included study. Microbiological failure, 90-day resistance development, and the 30-day mortality rates were set as the secondary outcomes. Screening of titles and abstracts of the retrieved records was performed independently by 2 authors (M. G. and R. D. P.). Potential discrepancies were resolved by means of discussion between the 2 authors.

### Data Extraction

Relevant data of each included study were extracted independently by 2 authors (R. D. P. and B. G.). Specifically, the following information was collected: study authors and year of publication, study characteristics (study design and country), funding, demographic features (age and sex), clinical severity of patients’ condition (ie, underlying comorbid conditions, Sequential Organ Failure Assessment score, intensive care unit admission, need for mechanical ventilation, and requirement for vasopressors or continuous renal replacement therapy), site/type of infection, β-lactam administration mode and daily dosing regimens, treatment duration, monotherapy or combination therapy, and outcome data. Corresponding authors of included studies reporting unclear and/or missing data were contacted for clarification.

### Risk of Bias Assessment

Two authors (R. D. P. and B. G.) assessed independently the risk of bias of the included studies in regard to the primary outcome. The Cochrane risk of bias (RoB 2.0) tool [17] and the Risk of Bias in Non-randomized Studies of Interventions (ROBINS-I) tool [18] were adopted for assessing RCTs and observational studies, respectively. Any disagreement between the 2 was discussed with a third author (M. G.).

### Meta-analysis

Both the primary and secondary outcomes retrieved from the included studies were meta-analyzed by pooling the adjusted odds ratios (ORs) of propensity score models, matched cohorts, or multivariate logistic regression analyses comparing ceftolozane-tazobactam and ceftazidime-avibactam and providing an adjustment for confounders. The treatment effects were expressed as ORs and 95% confidence intervals (CIs) for dichotomous data by using a random-effects model with an inverse variance method. Statistical significance was assessed by means of a *Z* test, defining as significant differences with *P* values <.05.

Statistical heterogeneity among studies was assessed by means of χ^2^ tests (with *P* < .10 indicating significant heterogeneity) and *I*^2^ tests (with >50% indicating substantial heterogeneity). Publication bias was assessed by visually inspecting the funnel plot of the Egger test [19]. Subgroup analyses based on the site/type of infection and on the β-lactam administration mode (intermittent infusion [II] vs extended infusion [EI] or continuous infusion) were also performed. Sensitivity analysis was carried out by excluding studies at high or serious/critical risk of bias. Statistical analysis was performed using of MedCalc for Windows statistical software (version 19.6.1).

## RESULTS

### Literature Search

**Figure 1.**
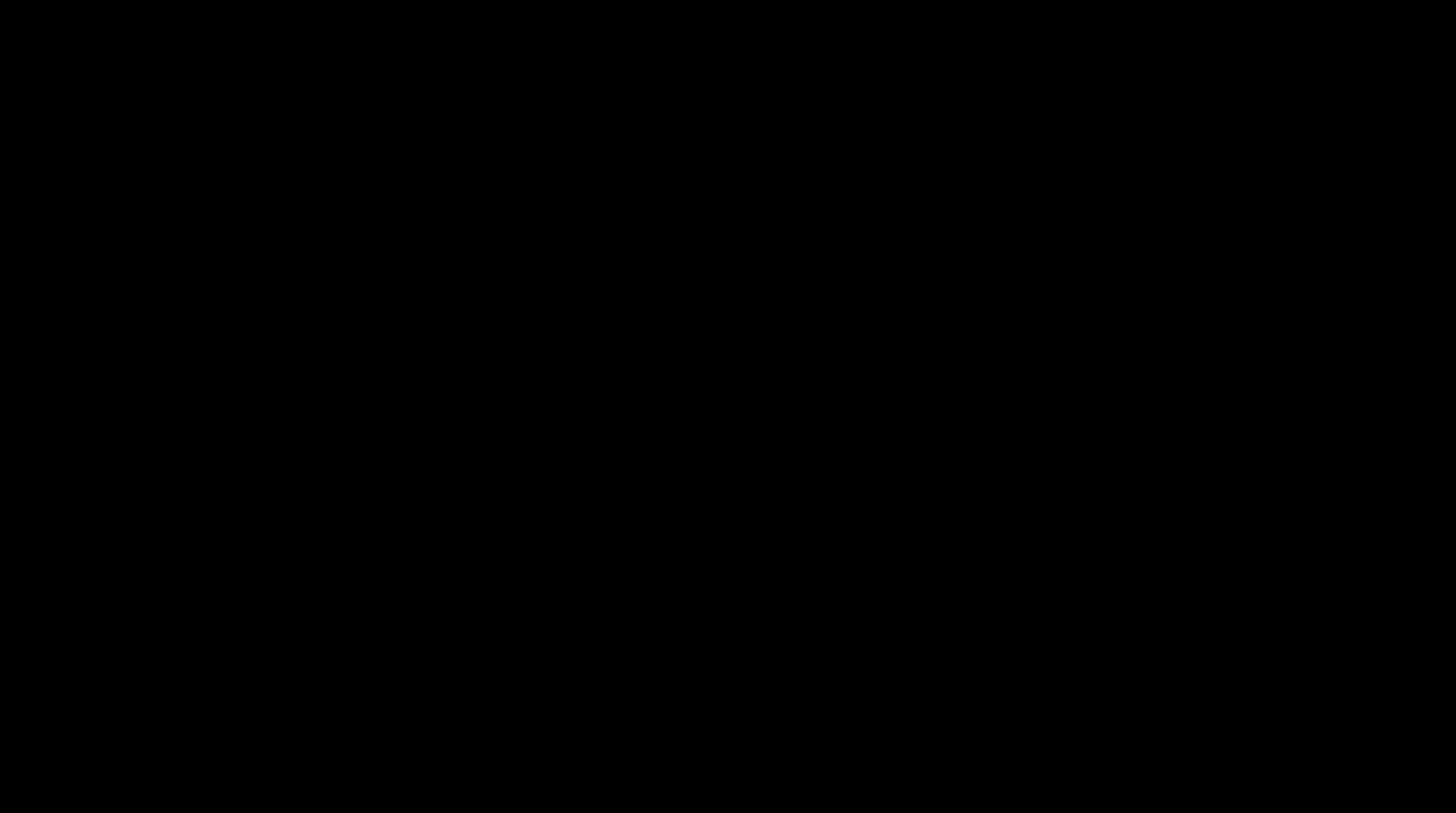
Preferred Reporting Items for Systematic Review and Meta-Analyses (PRISMA) flow diagram for study selection.

A total of 1193 potential studies was retrieved, of which 308 were removed as duplicates. Among the remaining 885 studies, 877 were excluded after screening of titles and abstracts. Overall, 8 full-text articles were assessed for eligibility, and finally 6 studies met the inclusion criteria, since 2 were excluded, one lacking an adjusted analysis and the other lacking a comparator group (Figure 1).

### Features of the Included Studies

All 6 included studies were observational (Table 1). One was prospective [22] and 5 retrospective [20, 21, 23–25], 4 were multicentric [20, 22, 23, 25], and 2 were monocentric [21, 24]. Four were conducted in the United States [22–25], and 1 each in Saudi Arabia [20] and the United Arab Emirates [21]. Overall, 1161 patients with documented MDR/DTR *P. aeruginosa* infections were enrolled (644 receiving ceftolozane-tazobactam and 517 receiving ceftazidime-avibactam). The *P. aeruginosa* status was defined as MDR based on Magiorakos et al [4] in 5 studies [20, 21, 23–25], and as DTR based on Kadri et al [5] in 1 [22].

**Table 1.** Main Features of Included Studies

Study Authors (Year) [Reference]	Study Design	Country	Time Period	Enrolled Patients, Total (C/T vs C/A), No.	Age, Median (IQR), C/T vs C/A, y^a^	Male Sex, C/T vs C/A, No. (%)	Comorbid Conditions and Severity Index, C/T vs C/A	Site/Type of Infection, C/T vs C/A	β-Lactam Administration Mode and Dosing Regimens	Treatment Duration, Median (IQR), C/T vs C/A, d^a^	Combination Therapy, C/T vs C/A	Definition of MDR/DTR *Pseudomonas aeruginosa*
Almangour et al (2023) [20]	Retrospective multicenter cohort study	Saudi Arabia	Jan 2017–Jun 2022	200 (100 vs 100)	Mean (SD), 60 (20) vs 60 (18)	71 (71) vs 61 (61)	Median CCI, 5 vs 5; hypertension, 53% vs 60%; DM, 57% vs 59% CVA, 20% vs 24%; CHF, 19% vs 20%; CAD, 18% vs 12%; liver disease, 9% vs 4%; COPD 5% vs 4%; immunosuppression, 33% vs 37%; APACHE score, 20% vs 19%; ICU admission, 25% vs 24%; AKI, 23% vs 17%; RRT, 4% vs 1%	HAP/VAP, 49%; SSTI, 24%; UTI, 10%; IAI, 8%; CR-BSI, 4%; other, 5% (referred to both agents)	C/T over 1 h, 100%; C/A over 2 h, 100%; C/T, 1.5 g every 8 h (other than pneumonia indications); 3 g every 8 h (pneumonia), adjusted according to renal function; C/A, 2.5 g every 8 h, adjusted according to renal function; mean/median daily dosing not reported	10 (7–15) vs 10 (6–14)	47% vs 35%	MDR based on classification system of Magiorakos et al [4]
El Nekidy et al (2024) [21]	Retrospective single-center cohort	United Arab Emirates	Sep 2018–Mar 2022	45 (20 vs 25)	69 (52–81) vs 69 (61.5–83)	7 (35) vs 9 (36)	Hypertension, 70% vs 76%; DM, 70% vs 64%; CAD, 40% vs 40%; CHF, 30% vs 24%; CVA, 55% vs 36%; respiratory failure, 75% vs 84%; ICU admission, 70% vs 72%; RRT, 100% vs 100%; VPs, 35% vs 32%; MV, 50% vs 52%	HAP/VAP, 85% vs 76%; BSI, 15% vs 24%	C/T over 1 h, 100%; C/A over 2 h, 100%; C/T, 1.5 g every 8 h (CRRT) or 0.75 g every 8 h (IHD); median (IQR) daily dosing, 2.25 g (1.5–2.25) g; C/A, 1.25 g every 8 h (CRRT) or 1.25 g/d (IHD); median (IQR) daily dosing, 1.88 (0.94–3.75) g	7 (4–11.5) vs 6 (4–8)	70% vs 60%	MDR based on classification system of Magiorakos et al [4]
Hareza et al (2024) [22]	Prospective observational cohort	United States	Jan 2018–Dec 2023	186 (102 vs 84)	59 (47–66) vs 59 (42–69)	57 (55.8) vs 45 (53.5)	CCI ≥5, 48.0% vs 48.8%; CKD, 40.2% vs 35.7%; DM, 34.3% vs 41.7%; CAD, 30.4% vs 17.9%; CVA, 10.8% vs 13.1%; liver disease 9.8% vs 7.1%; immunosuppression, 42.2% vs 23.8%^b^; ICU admission, 55.9% vs 60.7%; CRRT, 28.4% vs 26.2%; VPs, 40.2% vs 47.6%; MV, 53.9% vs 56%	HAP/VAP, 56.9% vs 53.6%; SSTI, 16.7% vs 13.1%; BSI, 13.7% vs 13.1%; UTI, 4.9% vs 13.1%; IAI, 4.9% vs 3.6%	1-h C/T, 38%; 3-h C/T, 62%; 2-h C/A, 100%; C/T: 3 g every 8 h, 73%; 1.5 g every 8 h, 27%, adjusted according to renal function; C/A, 2.5 g every 8 h, adjusted according to renal function; mean/median daily dosing not reported	11 (8–18) vs 10 (7–16)	12.8% vs 14.3%	DTR nonsusceptibility to all “traditional” β-lactams (ie, piperacillin-tazobactam, ceftazidime, cefepime, aztreonam, meropenem, and imipenem) and fluoroquinolones (ie, ciprofloxacin and levofloxacin) [5]
Lodise et al (2024) [23]	Retrospective multicenter cohort	United States	Jan 2016—Sep 2022 (excluding Mar–Dec 2020 for COVID period)	197 (117 vs 80)	Mean, 60.9 vs 60.4	82 (70.1) vs 50 (62.5)	Median CCI, 3 vs 4; COPD, 41.9% vs 42.5%; CHF, 41.9% vs 36.3%; renal disease, 34.2% vs 45.0%; DM, 20.5% vs 31.3%; CVA, 17.1% vs 20.0%; CAD, 14.5% vs 13.8%; liver disease, 2.6% vs 1.3%; ICU admission, 62.4% vs 70%; MV, 57.3% vs 71.3%; RRT, NA	HAP/VAP, 100% (referred to both agents)	NA	NA	≥2 d with an aminoglycoside: 31 (26.5%) vs 19 (23.8%); ≥2 d with a polymyxin: 14 (12%) vs 9 (11.3%)	MDR based on classification system of Magiorakos [4]
Shah et al (2024) [24]	Retrospective single-center study	United States	2019–2021	113 (93 vs 20)	66 (19–91) vs 64 (31–86)	58 (62) vs 13 (65)	Median CCI, 5 vs 5; transplant recipient, 27% vs 20%; SOFA score, 7% vs 7.5%; APACHE score, 22% vs 22.5%; MV, 68% vs 15%; RRT, NA	HAP/VAP, 84%; BSI, 16% (referred to both agents)	C/T: EI ≥3 h, 20%^b^; II over 1 h, 80%; C/A: EI, ≥3 h, 45%^b^; II over 2 h, 55%; C/T: 3 g every 8 h, 83%; C/A: 2.5 g every 8 h, 90%; mean/median daily dosing not reported	Median 13 (range, 2–138) vs 11.5 (2–26)	67% vs 30%	MDR based on classification system of Magiorakos et al [4]
Shields et al (2025) [25]	Multicenter, retrospective observational study	United States	Jan 2016–Dec 2023	420 (210 vs 210)	63 (51–70) vs 60 (49–69)	133 (63) vs 134 (64)	Median CCI, 5 vs 4; COPD, 35% vs 35%; DM, 33% vs 32%; CKD, 26% vs 23%; SOT recipient, 20% vs 15%; immunosuppression, 33% vs 24%; ICU admission, 81% vs 79%; median (IQR) SOFA score, 7 (4–10) vs 7 (4–10); RRT, 30% vs 30%; VPs, 40% vs 40%; MV, 71% vs 70%	HAP/VAP, 83% vs 83%; BSI, 17% vs 17%	C/T: EI ≥3 h, 31%^b^; II over 1 h, 69%; C/A: EI ≥3 h, 21%^b^; II over 2 h, 79%; 15% of patients in C/T group received a suboptimal dose for HAP/VAP (1.5 g every 8 h instead of 3 g every 8 h); mean/median daily dosing not reported	9.1 (6.3–13.3) vs 8.1 (6.1–12.6)	28% vs 27%	MDR based on classification system of Magiorakos et al [4]

Abbreviations: AKI, acute kidney injury; APACHE, Acute Physiology and Chronic Health Evaluation; BSI, bloodstream infection; C/A, ceftazidime-avibactam; CAD, coronary artery disease; CCI, Charlson Comorbidity Index; CHF, congestive heart failure; CKD, chronic kidney disease; COPD, chronic obstructive pulmonary disease; CR-BSI, catheter-related BSI; CRRT, continuous renal replacement therapy; C/T, ceftolozane-tazobactam; CVA, cerebrovascular accident; DM, diabetes mellitus; DTR, difficult-to-treat resistant; EI, extended infusion; HAP, hospital-acquired pneumonia; IAI, intrabdominal infection; ICU, intensive care unit; IHD, intermittent hemodialysis; II, intermittent infusion; IQR, interquartile range; MDR, multidrug-resistant; MV, mechanical ventilation; NA, not assessed; RRT, renal replacement therapy; SOFA, Sequential Organ Failure Assessment; SOT, solid organ transplant; SSTI, skin and soft-tissue infection; UTI, urinary tract infection; VAP, ventilator-associated pneumonia; VPs, vasopressors.

^a^Age and treatment duration area given as median (IQR) except where otherwise indicated.

^b^ *P* value <.05 in the comparison between C/T and C/A.

The demographics and clinical features of included studies are detailed in Table 1 and the Supplementary materials. No significant differences emerged between ceftolozane-tazobactam and ceftazidime-avibactam in terms of demographics, underlying comorbid conditions, the severity of clinical conditions, or the infection site/type, except for a higher prevalence of immunosuppressed patients among those receiving ceftolozane-tazobactam in the study by Hareza et al [22] (Table 1). Pneumonia represented the most frequent infection in all of the included studies (49%–100%), followed by BSI (4%–24%).

### Meta-analysis of the Primary and of Secondary Outcomes

**Figure 2.**
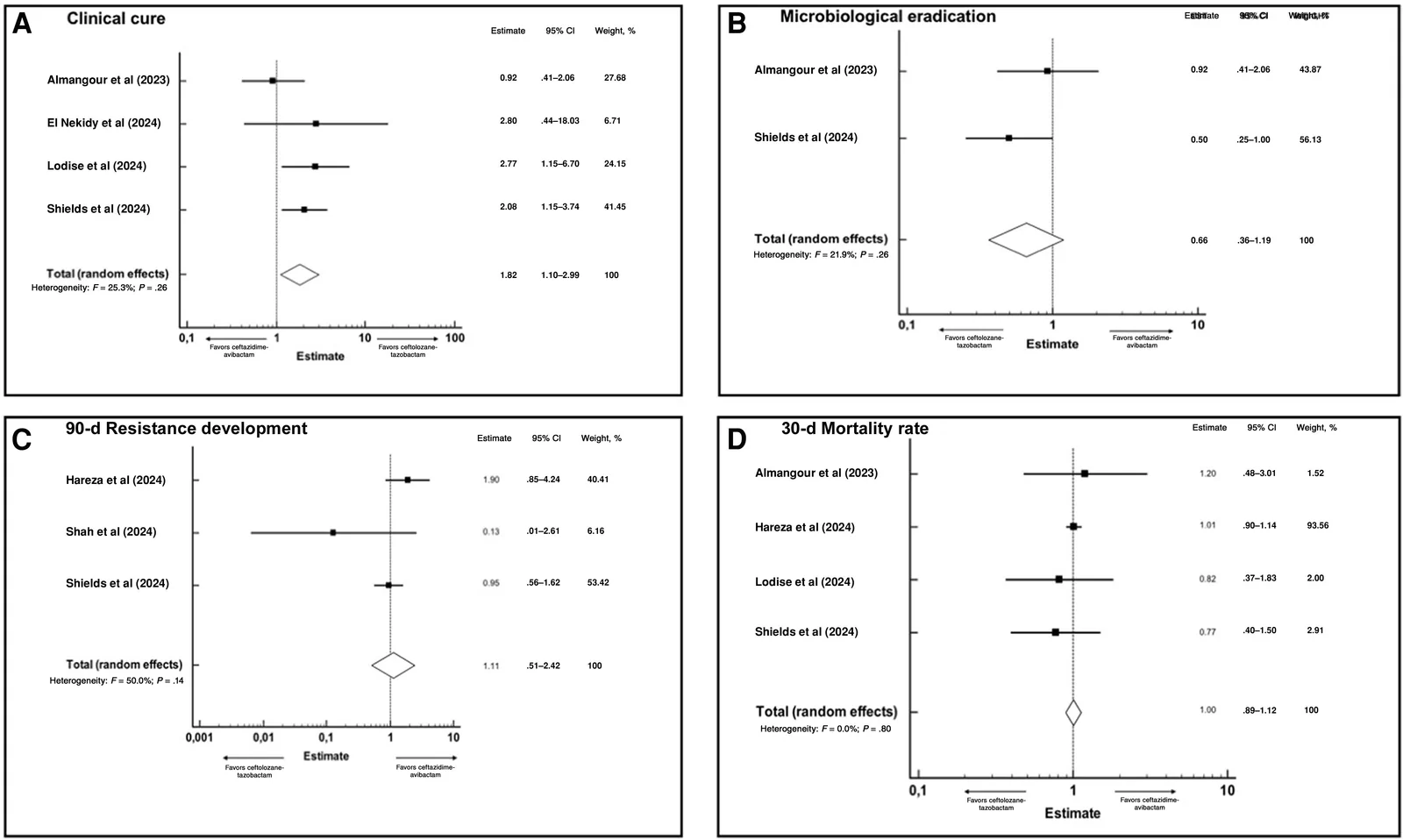
Forest plots of adjusted odds ratios showing clinical cure rate (*A*), microbiological failure rate (*B*), 90-day resistance development (*C*), and 30-day mortality rate (*D*) in patients treated with ceftolozane-tazobactam versus ceftazidime-avibactam for documented multidrug-resistant or difficult-to-treat resistant *Pseudomonas aeruginosa* infections [20–25]. Abbreviation: CI, confidence interval.

The definitions of both primary and secondary outcomes and the type of adjusted analysis provided in each of the included studies are summarized in Table 2. The forest plot of the meta-analyses concerning the primary and secondary outcomes are shown in Figure 2, and the corresponding findings are summarized in Table 3.

**Table 2.** Summary of Outcome Definitions for Each Included Study

Study Authors (Year) [Reference]	Clinical Cure	Resistance Occurrence	Microbiological Failure	Mortality Rate
Almangour et al (2023) [20]	Clinical cure was defined as resolution of infection manifestations after use of study drug, without a need for therapy to be modified due to failure or toxicity; clinical failure was defined as persistence of infection manifestations, despite adequate antimicrobial therapy; clinical, laboratory, and radiological findings were used to evaluate clinical outcomes, including fever, pulse rate, white blood cell counts, arterial blood gas, CRP, and procalcitonin levels, and imaging studies, when applicable; assessed by unblinded investigators at the end of treatment; adjusted according to multivariate logistic regression analysis	NA	Defined as persistence of positive cultures of etiological microorganisms at the same infection site, despite clinical outcome of infection (evaluated only if repeated cultures were available); assessed at 30 d; adjusted according to multivariate logistic regression analysis	Assessed at 30 d; adjusted according to multivariate logistic regression analysis
El Nekidy et al (2024) [21]	Clinical cure was defined as resolution of infection signs and symptoms, as reported in the treating physician's notes; clinical failure was defined as either persistence of functional symptoms for >72 h after initiation of the study drugs or failure documented by the treating physician, requiring a change in antimicrobials; efficacy parameters included clinical and microbiological clearance of bacteria, as well as changes in inflammatory markers; assessed at 30 d; adjusted according to multivariate logistic regression analysis	NA	No adjusted data provided	No adjusted data provided
Hareza et al (2024) [22]	NA	Defined as ≥4-fold rise in MIC of the antibiotic administered for the index infection; assessed at 90 d; adjusted according to inverse probability weighting using propensity score according to baseline patient and microbial characteristics	NA	Assessed at 30 d; adjusted according to inverse probability weighting using propensity score according to baseline patient and microbial characteristics
Lodise et al (2024) [23]	Defined as absence of recurrent MDR *Pseudomonas aeruginosa* pneumonia during index hospitalization and/or hospital readmission due to pneumonia/sepsis at 30 d; assessed at 30 d; adjusted according to multivariate logistic regression analysis	NA	NA	Assessed at 30 d; adjusted according to multivariate logistic regression analysis
Shah et al (2024) [24]	NA	Defined as MIC increased ≥4-fold from baseline; assessed at 90 d; adjusted according to inverse probability weighting using propensity score based on receipt of combination therapy, prolonged infusion, severity of illness, and prior cefepime exposure	No adjusted data provided	No adjusted data provided
Shields et al (2025) [25]	Defined as survival, resolution of signs and symptoms of infection with the intended treatment course, and absence of recurrent infection due to *P. aeruginosa*; assessed by unblinded investigators at 30 d; adjusted according to logistic regression analyses performed after 1:1 matching of the 2 cohorts, with adjustment for clinically relevant variables, including those disproportionately associated with 1 treatment group or outcome based on modified disjunctive cause criterion	Defined as isolation of any subsequent isolate demonstrating nonsusceptibility to study drug after ≥72 h of treatment as defined by CLSI breakpoints; assessed at 90 d; adjusted according to logistic regression analyses performed after 1:1 matching of the 2 cohorts, with adjustment for clinically relevant variables, including those disproportionately associated with 1 treatment group or outcome based on modified disjunctive cause criterion	Defined as recurrence of infection with or without resistance development; assessed at 90 d; adjusted according to logistic regression analyses performed after 1:1 matching of the 2 cohort, with adjustment for clinically relevant variables, including those disproportionately associated with 1 treatment group or outcome based on modified disjunctive cause criterion	Assessed at 30 d; adjusted according to logistic regression analyses performed after 1:1 matching of the 2 cohorts, with adjustment for clinically relevant variables, including those disproportionately associated with 1 treatment group or outcome based on modified disjunctive cause criterion

Abbreviations: CLSI, Clinical and Laboratory Standards Institute; CRP, C-reactive protein; MDR, multidrug-resistant; MIC, minimum inhibitory concentration; NA, not assessed.

**Table 3.** Meta-analysis of Primary and Secondary Outcomes in Patients With Multidrug-Resistant and/or Difficult-to-Treat Resistant *Pseudomonas aeruginosa* Treated With Ceftolozane-Tazobactam Versus Ceftazidime-Avibactam

Outcome	Studies, No.	Patients, No. (Ceftolozane-Tazobactam vs Ceftazidime-Avibactam)	OR (95% CI); *P* Value	Heterogeneity, *I*^2^, %; *P* Value	Publication Bias, *P* Value (Egger Test)
Clinical cure	4	862 (452 vs 410)	1.82 (1.10–2.99); .02	25.3; .26	.87
Microbiological failure	2	620 (310 vs 310)	0.66 (.36–1.19); .16	21.9; .26	NA
90-d Resistance development	3	719 (402 vs 317)	1.11 (.51–2.42); .79	50.0; .14	.72
30-d Mortality rate	4	1003 (529 vs 474)	1.00 (.89–1.12); .99	0.0; .80	.50

Abbreviations: CI, confidence interval; NA, not assessed; OR, odds ratio.

Four studies provided data for assessing clinical cure (862 patients) [20, 21, 23, 25]. Overall, the clinical cure rate was higher with ceftolozane-tazobactam than with ceftazidime-avibactam in the treatment of documented MDR/DTR *P. aeruginosa* infections (OR, 1.82 [95% CI, 1.10–2.99]; *P* = .02) (Figure 2*A*). Neither the degree of heterogeneity (*I*^2^ = 25.3%; *P* = .26) nor the publication bias (*P* = .87) showed significant differences.

Two studies provided data for assessing microbiological failure (620 patients) [20, 25]. Overall, no significant difference emerged between the use of ceftolozane-tazobactam and of ceftazidime-avibactam (OR, 0.66 [95% CI, .36–1.19]; *P* = .16) (Figure 2*B*). The degree of heterogeneity was not significant (*I*^2^ = 21.9%; *P* = .26).

Three studies provided data for assessing the 90-day resistance occurrence (719 patients) [22, 24, 25]. Overall, no significant difference emerged between ceftolozane-tazobactam and ceftazidime-avibactam (OR, 1.11 [95% CI, .51–2.42]; *P* = .79) (Figure 2*C*). The degree of heterogeneity (*I*^2^ = 50.0%; *P* = .14) and the publication bias (*P* = .72) were not significant.

Four studies provided data for assessing the 30-day mortality rate (1003 patients) [20, 22, 23, 25]. Overall, no significant difference was reported between ceftolozane-tazobactam and ceftazidime-avibactam (OR, 1.00 [95% CI, .89–1.12]; *P* = .99) (Figure 2*D*). The degree of heterogeneity (*I*^2^ = 0.0%; *P* = .80) and the publication bias (*P* = .50) were not significant.

### Subgroup and Sensitivity Analyses

Two studies provided adjusted data for the primary outcome and for the 30-day mortality rate in the subgroup of patients affected by pneumonia (547 patients) [23, 25]. Overall, ceftolozane-tazobactam was associated with a higher clinical cure rate than ceftazidime-avibactam (OR, 2.48 [95% CI, 1.47–4.18]; *I*^2^ = 0.0%), but no difference was seen in the 30-day mortality rate (OR, 0.90 [.54–1.52]; *I*^2^ = 0.0%).

Two studies included patients with antimicrobial therapy administered via II [20, 21]. The subgroup meta-analysis showed no significant impact of this modality on clinical cure differences between the 2 agents (OR, 1.16 [95% CI, .48–2.77]; *I*^2^ = 13.1%). Meta-analyses of the secondary outcomes were not feasible since only 1 study provided adjusted outcome data for microbiological failure or the mortality rate [20].

Two studies included patients in which antimicrobial therapies were administered via EI [24, 25]. The subgroup meta-analysis showed no significant impact of this modality on differences in 90-day resistance between the 2 agents (OR, 0.62 [95% CI, .13–3.08]; *I*^2^ = 39.1%). Meta-analyses of the primary and of the other secondary outcomes were not feasible since only 1 study provided adjusted data for these outcomes [25].

Sensitivity analysis for both the primary outcome and microbiological failure was not feasible given that only 1 study was at moderate risk of bias in this regard [25]. Regarding the other secondary outcomes, no significant difference emerged between 90-day resistance occurrence and 30-day mortality rate compared with the primary analysis, after exclusion of studies at serious and/or critical risk of bias.

### Quality of the Included Studies

Three of the 6 included studies were at moderate risk of bias, and the other 3 were at serious risk of bias in ≥1 domain, with confounders being the most frequent bias (Supplementary Table 1).

## DISCUSSION

To the best of our knowledge, this is the first meta-analysis comparing the clinical efficacy of ceftolozane-tazobactam and ceftazidime-avibactam in the treatment of hospitalized patients affected by MDR/DTR *P. aeruginosa* infections after adjustment for confounders. Notably, our findings showed that in this challenging scenario ceftolozane-tazobactam could enable a higher clinical cure rate than ceftazidime-avibactam, whereas microbiological outcome, 90-day resistance development, and mortality rates did not differ between the 2 agents.

Regarding the primary outcome, it may be speculated that some peculiar pharmacological differences between the 2 agents could explain the better clinical cure rate with ceftolozane-tazobactam in the management of MDR/DTR *P. aeruginosa* infections. Looking at the antipseudomonal activity, ceftolozane-tazobactam may exhibit wider coverage against MDR/DTR *P. aeruginosa* strains than ceftazidime-avibactam. In fact, ceftolozane only, by being stable to AmpC degradation and at the same time by crossing the outer membrane irrespective of the expression of the OprD porin, retains valuable activity against strains either overexpressing AmpC or defecting OprD porin [3, 13, 14]. Looking at the PK/PD profile, it could be expected that optimal PK/PD target attainment against MDR/DTR *P. aeruginosa* could be more likely with standard dosing regimens for ceftolozane-tazobactam than with ceftazidime-avibactam. In fact, it should not be overlooked that the activity of ceftolozane-tazobactam against MDR/DTR *P. aeruginosa* depends only on ceftolozane and is completely unaffected by tazobactam [11]. Conversely, the activity of ceftazidime-avibactam depends strictly on avibactam’s restoring of susceptibility to ceftazidime [11]. Consequently, the likelihood of optimal joint PK/PD target attainment with ceftazidime-avibactam may be affected whenever a patient’s underlying pathophysiological conditions alters the balance between ceftazidime and avibactam concentrations.

Notably, a retrospective observational study carried out among critically ill patients receiving ceftazidime-avibactam by continuous infusion showed that in patients without renal dysfunction avibactam clearance may be much faster than ceftazidime clearance, so that an optimal joint PK/PD target could be more difficult to be attained in these cases [15]. Specifically, patients with a creatinine clearance >80 mL/min/1.73 m^2^ showed significantly greater proportions of ceftazidime-to-avibactam ratios >5:1 (59.3% vs 23.8%; *P* < .001) and >6:1 (32.1% vs 6.3%; *P* < .001) than patients with mild-to-severe renal dysfunction and a creatinine clearance <80 mL/min/1.73 m^2^ [15]. Attaining an optimal joint PK/PD target with ceftazidime-avibactam could be even more difficult than with ceftolozane alone when treating hospital-acquired or ventilator-associated pneumonia with standard dosing regimens delivered via II, namely 2 conditions shared by the vast majority of the patients included in this meta-analysis [20–25]. On the one hand, in fact, II administration may result in significantly lower plasma trough levels than continuous infusion, and on the other hand, the higher penetration rate of ceftolozane in the epithelial lining fluid (about 50% [26]) compared with the penetration rates of both ceftazidime and avibactam (about 32%–35% [27]) may increase the likelihood of optimal PK/PD target attainment at this infection site with ceftolozane.

This could explain why the clinical cure rate in the pneumonia subgroup was found to be approximately 2.5-fold higher with ceftolozane-tazobactam than with ceftazidime-avibactam. Conversely, the subgroup analysis based on different administration modes did not show any difference between the 2 agents in terms of either clinical cure or 90-day resistance development. Indeed, it may be speculated that the absence of patients receiving continuous infusion in any of the included studies could have biased the findings. Previous studies showed that continuous infusion of ceftolozane-tazobactam, compared with both II and EI, was associated with higher bacterial burden reduction and better likelihood of aggressive PK/PD target attainment against MDR/DTR *P. aeruginosa* [28, 29].

The findings of our meta-analysis are in contrast with those of 2 others recently published studies [30, 31]. Almangour et al [30] meta-analyzed 10 observational studies and found no significant difference between ceftazidime-avibactam and ceftolozane-tazobactam in terms of clinical cure (OR, 0.94 [.61–1.43]). Likewise, Collings et al [31] meta-analyzed 16 observational studies (many comparing ceftolozane-tazobactam with aminoglycosides and/or with polymyxin and not with ceftazidime-avibactam) and did not find any difference between ceftazidime-avibactam and ceftolozane-tazobactam in terms of clinical cure (OR, 0.92; [95% CI, .57–1.60]) [31]. Indeed, the differences in clinical outcome between study and the 2 other meta-analyses could be at least partially explained by the presence of some potential methodological flaws affecting the other studies [30, 31]. Almangour et al [30] included 2 studies in which *P. aeruginosa* infections were caused either by MDR/DTR or multisusceptible clinical isolates. The authors pooled crude unadjusted data to estimate the eventual association effect of ceftolozane-tazobactam with the different selected outcomes, a practice strongly discouraged by the Cochrane group for analyses of observational studies because they are prone to multiple biases and confounding factors [32]. Both of the other meta-analyses [30, 31] also included studies that were excluded in ours according to the selection criteria, namely, those with unadjusted effect estimates for the different selected outcomes. In addition, these studies did not adopt the inverse variance method for pooling adjusted effect estimates.

The current meta-analysis did not show any significant difference between the 2 agents in terms of secondary outcomes, namely, microbiological outcome and 90-day resistance development. The prevalence rates of resistance development to both ceftolozane-tazobactam and ceftazidime-avibactam in 3 of the included studies were quite high (22% and 23%, respectively, in the study of Shields et al [25], 10% and 40% in that of Shah et al [24], 38% and 25% in that of Hareza et al [22]). In this regard, it should not be overlooked that the vast majority of the patients included in this meta-analysis received both agents as standard dosing regimens (namely 3 g every 8 hours for ceftolozane-tazobactam over 1 hour and 2.5 g every 8 hours over 2 hours for ceftazidime-avibactam, adapted for renal dysfunction) administered by II. This dosing regimen was shown to suffice for attaining the conventional PK/PD target needed for clinical efficacy (ie, 50%–100% of the time with β-lactam concentrations persisting above the minimum inhibitory concentration [MIC]). However, it could be insufficient for attaining the aggressive PK/PD target needed to prevent resistance development (ie, 100% of the time with β-lactam concentrations persisting at >4-fold the MIC) [12].

It is noteworthy that a recent meta-analysis showed that attaining an aggressive PK/PD target may be much more protective than attaining a conventional one when dealing with resistance development to β-lactams used to treat documented gram-negative infections in critically ill patients (OR, 0.06 [95% CI, .01–.29]; *P* < .001) [33]. Several studies have shown that continuous infusion administration may be a helpful strategy either for increasing the likelihood of aggressive PK/PD target attainment or for decreasing that of resistance development to ceftolozane-tazobactam [29, 34–36]. Specifically, regarding the former, in a prospective study including 72 patients receiving ceftolozane-tazobactam for the treatment of DTR *P. aeruginosa* infections, continuous infusion was shown be more effective than both II and EI in attaining the aggressive PK/PD target in borderline-susceptible isolates with an MIC ≥2 mg/L [29]. In a case-control study in 28 patients receiving ceftolozane-tazobactam for the treatment of DTR *P. aeruginosa* infections, EI (over 3 hours) was significantly associated with a lower resistance development rate than II (0.0% vs 58.3%; *P* = .04) [34].

A population PK model conducted among 88 critically ill patients receiving ceftolozane-tazobactam by continuous infusion showed that this approach always allowed aggressive PK/PD target attainment across all the kidney function strata against *P. aeruginosa* isolates exhibiting MICs up to the EUCAST clinical breakpoint of 4 mg/L [35]. Regarding the latter, a prepost quasi-experimental study carried in 85 patients with *P. aeruginosa* BSIs and/or ventilator-associated pneumonia treated with continuous infusion ceftolozane-tazobactam monotherapy found that an aggressive PK/PD target was attain in all patients in the postintervention phase by means of a therapeutic drug monitoring–guided strategy and that 30-day resistance development was almost halved compared with the preintervention phase, although this difference was not significant (10.8% vs 18.8%; *P* = .37) [36].

Overall, based on the findings of our meta-analysis, it may be speculated that, with standard dosing regimens of ceftolozane-tazobactam and ceftazidime-avibactam delivered by II to treat MDR/DTR *P. aeruginosa*, ceftolozane-tazobactam could attain better clinical cure compared with ceftazidime-avibactam, especially in patients with pneumonia and without renal dysfunction.

The limitations of our meta-analysis should be recognized. All but 1 of the included studies comparing the 2 agents had a retrospective design, and no RCT was retrieved. None of the included studies was at low risk of bias. The primary outcome was assessed based on only 4 studies, 3 of which were at serious risk of bias. Although recall bias could not be ruled out, none of the included studies was at serious or critical risk for missing outcome data since the available data were exhaustive. It may be challenging to extrapolate the emerged findings to types of infection other than pneumonia. Sensitivity analysis for the primary outcome was not feasible considering that only 1 study showed a moderate risk of bias. Finally, the unavailability of studies conducted in Europe could possibly limit the generalizability of the findings in settings with a different *P. aeruginosa* epidemiology in terms of susceptibility to these agents. Conversely, including only studies adjusted for confounders may represent a strength of our analysis, by minimizing the potential risk of bias.

In conclusion, our meta-analysis showed that, after appropriate adjustment for confounders, ceftolozane-tazobactam enabled a higher clinical cure rate in the treatment of MDR/DTR *P. aeruginosa* infections compared with ceftazidime-avibactam. RCTs as well as prospective studies assessing the relationship between aggressive PK/PD target attainment and clinical outcome in MDR/DTR *P. aeruginosa* infections would be warranted to hopefully confirm our findings and provide a definitive answer to this unmet clinical need. Meanwhile, the findings could help clinicians facing this challenging scenario, especially in patients with pneumonia and without renal dysfunction having.

## Supplementary Material

ciag194_Supplementary_Data

## Supplementary Data

Supplementary materials are available at *Clinical Infectious Diseases* online. Consisting of data provided by the authors to benefit the reader, the posted materials are not copyedited and are the sole responsibility of the authors, so questions or comments should be addressed to the corresponding author.

## References

[1] Horcajada JP, Montero M, Oliver A, et al Epidemiology and treatment of multidrug-resistant and extensively drug-resistant *Pseudomonas aeruginosa* infections. Clin Microbiol Rev 2019; 32:e00031-19. 10.1128/CMR.00031-19 PMC6730496 31462403

[2] Horcajada JP, Gales AC, Isler B, et al Current and future options for the treatment of serious infections due to carbapenem-resistant *Pseudomonas aeruginosa*. Clin Microbiol Rev 2025;38:e00233-24. 10.1128/cmr.00233-24 PMC12697150 41065368

[3] Horcajada JP, Gales A, Isler B, et al How do I manage difficult-to-treat *Pseudomonas aeruginosa* infections? Key questions for today's clinicians. Clin Microbiol Infect 2025; 31:1797–806. 10.1016/j.cmi.2025.08.018 40854462

[4] Magiorakos AP, Srinivasan A, Carey RB, et al Multidrug-resistant, extensively drug-resistant and pandrug-resistant bacteria: an international expert proposal for interim standard definitions for acquired resistance. Clin Microbiol Infect 2012; 18:268–81. 10.1111/j.1469-0691.2011.03570.x 21793988

[5] Kadri SS, Adjemian J, Lai YL, et al Difficult-to-treat resistance in gram-negative bacteremia at 173 US hospitals: retrospective cohort analysis of prevalence, predictors, and outcome of resistance to all first-line agents. Clin Infect Dis 2018; 67:1803–14. 10.1093/cid/ciy378 PMC6260171 30052813

[6] Cosentino F, Viale P, Giannella M. MDR/XDR/PDR or DTR? Which definition best fits the resistance profile of *Pseudomonas aeruginosa*? Curr Opin Infect Dis 2023; 36:564–71. 10.1097/QCO.0000000000000966 PMC10836784 37930070

[7] Yahav D, Giske CG, Grāmatniece A, Abodakpi H, Tam VH, Leibovici L. New β-lactam-β-lactamase inhibitor combinations. Clin Microbiol Rev 2020; 34:e00115-10.1128/CMR.00115-20 PMC7667665 33177185

[8] Sader HS, Mendes RE, Arends SJR, Carvalhaes CG, Shortridge D, Castanheira M. Comparative activity of newer β-lactam/β-lactamase inhibitor combinations against *Pseudomonas aeruginosa* isolates from US medical centres (2020–2021). Int J Antimicrob Agents 2023; 61:106744. 10.1016/j.ijantimicag.2023.106744 36738849

[9] Sader HS, Carvalhaes CG, Shortridge D, Castanheira M. Comparative activity of newer β-lactam/β-lactamase inhibitor combinations against *Pseudomonas aeruginosa* from patients hospitalized with pneumonia in European medical centers in 2020. Eur J Clin Microbiol Infect Dis 2022; 41:319–24. 10.1007/s10096-021-04363-7 PMC8770431 34657213

[10] Tamma PD, Heil EL, Justo JA, Mathers AJ, Satlin MJ, Bonomo RA. Infectious Diseases Society of America 2024 guidance on the treatment of antimicrobial-resistant gram-negative infections. Clin Infect Dis doi:10.1093/cid/ciae403. Published 7 August 2024. 39108079

[11] Bush K, Bradford PA. Interplay between β-lactamases and new β-lactamase inhibitors. Nat Rev Microbiol 2019; 17:295–306. 10.1038/s41579-019-0159-8 30837684

[12] Gatti M, Pea F. Jumping into the future: overcoming pharmacokinetic/pharmacodynamic hurdles to optimize the treatment of severe difficult to treat-gram-negative infections with novel beta-lactams. Expert Rev Anti Infect Ther 2023; 21:149–66. 10.1080/14787210.2023.2169131 36655779

[13] Takeda S, Nakai T, Wakai Y, Ikeda F, Hatano K. In vitro and in vivo activities of a new cephalosporin, FR264205, against *Pseudomonas aeruginosa*. Antimicrob Agents Chemother 2007; 51:826–30. 10.1128/AAC.00860-06 PMC1803152 17145788

[14] Moyá B, Zamorano L, Juan C, Ge Y, Oliver A. Affinity of the new cephalosporin CXA-101 to penicillin-binding proteins of *Pseudomonas aeruginosa*. Antimicrob Agents Chemother 2010; 54:3933–7. 10.1128/AAC.00296-10 PMC2934984 20547785

[15] Gatti M, Viale P, Pea F. Therapeutic drug monitoring of ceftazidime/avibactam: why one leg is not enough to run. J Antimicrob Chemother 2024; 79:195–9. 10.1093/jac/dkad367 38019676

[16] Page MJ, McKenzie JE, Bossuyt PM, et al The PRISMA 2020 statement: an updated guideline for reporting systematic reviews. BMJ 2021;372:n71. 10.1136/bmj.n71 PMC8005924 33782057

[17] Sterne JAC, Savović J, Page MJ, et al Rob 2: a revised tool for assessing risk of bias in randomised trials. BMJ 2019; 366:l4898. 10.1136/bmj.l4898 31462531

[18] Sterne JA, Hernán MA, Reeves BC, et al ROBINS-I: a tool for assessing risk of bias in non-randomised studies of interventions. BMJ 2016; 355:i4919. 10.1136/bmj.i4919 PMC5062054 27733354

[19] Egger M, Smith GD, Schneider M, Minder C. Bias in meta-analysis detected by a simple, graphical test. BMJ 1997; 315:629–34. 10.1136/bmj.315.7109.629 PMC2127453 9310563

[20] Almangour TA, Ghonem L, Alassiri D, et al Ceftolozane-tazobactam versus ceftazidime-avibactam for the treatment of infections caused by multidrug-resistant *Pseudomonas aeruginosa*: a multicenter cohort study. Antimicrob Agents Chemother 2023; 67:e00405-23. 10.1128/aac.00405-23 PMC10433809 37404159

[21] El Nekidy WS, Al Ali M, Abidi E, et al Clinical outcomes of ceftazidime–avibactam versus ceftolozane–tazobactam in managing pseudomonal infections in patients undergoing renal replacement therapy. Antibiotics 2024; 13:699. 10.3390/antibiotics13080699 PMC11350821 39199999

[22] Hareza DA, Cosgrove SE, Bonomo RA, et al Clinical outcomes and emergence of resistance of *Pseudomonas aeruginosa* infections treated with ceftolozane-tazobactam versus ceftazidime-avibactam. Antimicrob Agents Chemother 2024; 68:e00907-24. 10.1128/aac.00907-24 PMC11459953 39230311

[23] Lodise TP, Obi EN, Watanabe AH, Yucel E, Min J, Nathanson BH. Comparative evaluation of early treatment with ceftolozane/tazobactam versus ceftazidime/avibactam for non-COVID-19 patients with pneumonia due to multidrug-resistant *Pseudomonas aeruginosa*. J Antimicrob Chemother 2024;79:2954–64. 10.1093/jac/dkae313 PMC11531822 39258877

[24] Shah S, Kline EG, Haidar G, et al Rates of resistance to ceftazidime-avibactam and ceftolozane-tazobactam among patients treated for multidrug-resistant *Pseudomonas aeruginosa* bacteremia or pneumonia. Clin Infect Dis 2025; 80:24–8. 10.1093/cid/ciae332 PMC12057557 38902935

[25] Shields RK, Abbo LM, Ackley R, et al Effectiveness of ceftazidime–avibactam versus ceftolozane–tazobactam for multidrug-resistant *Pseudomonas aeruginosa* infections in the USA (CACTUS): a multicentre, retrospective, observational study. Lancet Infect Dis 2025; 25:574–84. 10.1016/S1473-3099(24)00648-0 39701120

[26] Caro L, Nicolau DP, De Waele JJ, et al Lung penetration, bronchopulmonary pharmacokinetic/pharmacodynamic profile and safety of 3 g of ceftolozane/tazobactam administered to ventilated, critically ill patients with pneumonia. J Antimicrob Chemother 2020; 75:1546–53. 10.1093/jac/dkaa049 PMC7225904 32211756

[27] Dimelow R, Wright JG, MacPherson M, Newell P, Das S. Population pharmacokinetic modelling of ceftazidime and avibactam in the plasma and epithelial lining fluid of healthy volunteers. Drugs R D 2018; 18:221–30. 10.1007/s40268-018-0241-0 PMC6131119 30054895

[28] Montero MM, Domene-Ochoa S, López-Causapé C, et al Comparison of ceftolozane/tazobactam infusion regimens in a hollow-fiber infection model against extensively drug-resistant *Pseudomonas aeruginosa* isolates. Microbiol Spectr 2022; 10:e00892-22. 10.1128/spectrum.00892-22 PMC9241897 35695526

[29] Pilmis B, Petitjean G, Lesprit P, et al Continuous infusion of ceftolozane/tazobactam is associated with a higher probability of target attainment in patients infected with *Pseudomonas aeruginosa*. Eur J Clin Microbiol Infect Dis 2019; 38:1457–61. 10.1007/s10096-019-03573-4 31073653

[30] Almangour TA, Alzunaydi S, AlManea F, et al Ceftazidime-avibactam versus ceftolozane-tazobactam in the treatment of drug-resistant *Pseudomonas aeruginosa*: a meta-analysis. Int J Infect Dis 2026; 165:108485. 10.1016/j.ijid.2026.108485 41698495

[31] Collings H, Stone M, Chrysostomou M, et al A systematic literature review and (network) meta-analysis of the effectiveness of ceftolozane/tazobactam versus aminoglycosides/polymyxins and ceftazidime/avibactam for treating adult patients with multidrug-resistant *Pseudomonas aeruginosa* infections. Antimicrob Agents Chemother 2026; 70:e00735-25. 10.1128/aac.00735-25 PMC12888884 41532768

[32] Cochrane Handbook . Chapter 24: including non-randomized studies on intervention effects. Available at: https://www.cochrane.org/authors/handbooks-and-manuals/handbook/current/chapter-24. Accessed 25 February 2026.

[33] Gatti M, Cojutti PG, Pea F. Impact of attaining aggressive vs conservative PK/PD target on the clinical efficacy of beta-lactams for the treatment of gram-negative infections in the critically ill patients: a systematic review and meta-analysis. Crit Care 2024; 28:123. 10.1186/s13054-024-04911-5 PMC11020314 38627763

[34] Tamma PD, Beisken S, Bergman Y, et al Modifiable risk factors for the emergence of ceftolozane-tazobactam resistance. Clin Infect Dis 2021; 73:e4599–606. 10.1093/cid/ciaa1306 32881997

[35] Cojutti PG, Pai MP, Gatti M, et al Targeting *Pseudomonas aeruginosa* resistance at the exposure frontier: a population PK/PD blueprint for ceftolozane/tazobactam continuous infusion. Antimicrob Agents Chemother 2025; 69:e01042-25. 10.1128/aac.01042-25 PMC12587603 40980909

[36] Gatti M, Rinaldi M, Bonazzetti C, et al A pre-post quasi-experimental study of the impact of TDM-guided aggressive pharmacokinetic/pharmacodynamic target attainment of continuous infusion ceftolozane/tazobactam monotherapy in treating severe *Pseudomonas aeruginosa* infections: a strategy useful for raising the bar? J Antimicrob Chemother 2025; 80:1543–51. 10.1093/jac/dkaf098 PMC12129583 40126532

