# Supplementary material for: Comparison of Ceftolozane-Tazobactam and Ceftazidime-Avibactam in the Treatment of Multidrug-Resistant/Difficult-to-Treat Resistant Pseudomonas aeruginosa Infections: A Systematic Review and Meta-analysis
Source: Clin Infect Dis. 2026 Mar 18;83(2):e270–81. doi: 10.1093/cid/ciag194 (PMC13537137; doi:10.1093/cid/ciag194)
Supplement: Supplementary file 1 [file ciag194_supplementary_data.docx]

**Supplementary materials**

*Demographics and clinical features of included studies*

Patients had median and/or mean age ranging from 59 to 69 years, a male preponderance (ranging from 53.5% to 71.0%) in all but one studies [20,21,23–25] and an ICU admission higher than 50% in all of the studies [20,22–25] except that of Almangour *et al*. [21]. Data concerning the BL/BLIc administration mode were provided in all but one study [20–24]. Ceftolozane-tazobactam was administered by intermittent infusion (II) over 1 hour in all of the patients included in two studies [21,22] and in a proportion ranging from 38% to 80% of those included in the other three studies [20,23,24]. Extended infusion (EI) over at least 3 hours was adopted in the remaining proportions of patients of these three other studies, namely ranging from 20% to 62% [20,23,24]. Ceftazidime-avibactam was administered by II over 2 hours in all of the included patients of three studies, and in a proportion ranging from 55% to 79% of those included in the other two studies [23,24]. EI over at least 3 hours was adopted in the remaining proportion of patients of the two other studies, namely ranging from 21% to 45% [23,24]. Continuous infusion (CI) administration was never adopted in any study, neither for ceftolozane-tazobactam nor for ceftazidime-avibactam. Median treatment duration was similar in the two groups, ranging from 7 to 13 days for ceftolozane-tazobactam and from 6 to 11.5 days for ceftazidime-avibactam. The rate of combination therapy ranged from 12.8% to 70.0% in the ceftolozane-tazobactam cohort and from 14.3% to 60.0% in that of ceftazidime-avibactam.

**Supplementary Table 1 –** Risk of bias assessment for observational studies according to ROBINS-I tool

**Supplementary Table 1 –** Risk of bias assessment for observational studies according to ROBINS-I tool

| **Study** | **Bias domains** | | | | | | | **Overall judgment** |
| --- | --- | --- | --- | --- | --- | --- | --- | --- |
|  | **Bias due to confounding** | **Bias in selection of participants** | **Bias in classification of exposure** | **Bias due to deviations from intended exposure** | **Bias due to missing data** | **Bias in measurement of outcome** | **Bias in selection of reported result** |  |
| Almangour *et al.*, 2024 | Serious | Serious | Low | Moderate | Moderate | Moderate | Low | Serious |
| El Nekidy *et al.*, 2024 | Serious | Moderate | Low | Moderate | Moderate | Moderate | Moderate | Serious |
| Hareza *et al*., 2024 | Moderate | Moderate | Low | Moderate | Moderate | Moderate | Moderate | Moderate |
| Lodise *et al*., 2024 | Serious | Moderate | Low | Moderate | Moderate | Serious | Moderate | Serious |
| Shah *et al.*, 2024 | Moderate | Low | Low | Moderate | Low | Low | Low | Moderate |
| Shields *et al.*, 2025 | Moderate | Moderate | Low | Moderate | Low | Moderate | Low | Moderate |
